# Association of left atrial volume and function parameters with cardiovascular outcomes following kidney transplantation

**DOI:** 10.1186/s12947-026-00369-3

**Published:** 2026-03-02

**Authors:** Ava R. DeLonais-Parker, Spencer H. Hobbs, Taylor R. Coffman, Michael F. Kanan, Yanqing Lyu, Elsa J. Treiber, Rushda F. Mansuri, Barbara C. Okeke, Krista L. Lentine, Mina M. Benjamin

**Affiliations:** 1 Saint Louis University School of Medicine, Saint Louis, MO USA; 2 Department of Epidemiology and Biostatistics, College for Public Health and Social Justice, Saint Louis University, Saint Louis, MO USA; 3 Internal Medicine Department, Saint Louis University Hospital, Saint Louis, MO USA; 4 Division of Nephrology, Saint Louis University Hospital, Saint Louis, MO USA; 5 Division of Cardiology, Division of Cardiovascular Medicine, SSM-Saint Louis University Hospital, 1201 S Grand Blvd.,, Saint Louis, MO 63104 USA

**Keywords:** Echocardiography, Left atrial strain, Kidney transplant, Major adverse cardiovascular events

## Abstract

**Purpose:**

Left atrial (LA) volume and strain parameters have been associated with cardiovascular outcomes in several cardiac pathologies, yet their role in predicting major adverse cardiovascular events (MACE) in kidney transplant (KT) recipients has not been explored.

**Methods:**

We retrospectively reviewed the records of adult KT recipients from our institution (2015–2024). We utilized baseline echocardiograms routinely acquired during KT workup to measure LA volumetrics and strain. MACE was the study’s primary endpoint, defined as cardiovascular death, nonfatal myocardial infarction, stroke, major arrhythmias or heart failure hospitalization. Logistic regression, Kaplan-Meier and Cox proportional hazards regression were performed to evaluate the association between LA parameters and MACE.

**Results:**

Of 518 patients who underwent kidney transplant, 377 were in sinus rhythm with an acceptable quality echocardiogram (male, 56.7%; mean age 53.7 ± 13.1 years). Over a median follow up duration of 5.3 ± 2.3 years from KT, 82 patients reached the study endpoint. Kaplan-Meier analysis showed significantly lower MACE-free survival in patients with abnormal LA strain. After adjusting for confounding variables in the Cox Proportional Hazards model, of all LA parameters, lower LAScd (HR 0.94, 95% CI 0.89–0.98, *p* = 0.003), and LASr (HR 0.97, 95% CI 0.94–0.995, *p* = 0.02) were independently associated with MACE.

**Conclusion:**

In this retrospective single center study, LA strain parameters particularly LASr and LAScd were independently associated with MACE after KT. LA strain might have a role in risk stratification in this population.

**Supplementary Information:**

The online version contains supplementary material available at 10.1186/s12947-026-00369-3.

## Introduction

Cardiovascular disease (CVD) is the leading cause of morbidity and mortality among kidney transplant (KT) recipients [1, 2]. Although the number of KTs performed annually has continued to rise, surpassing 27,000 in 2024 and totaling over 600,000 since 1988 [3], approximately 90,000 individuals remain on the transplant waitlist at any given time [4]. The large number of vulnerable patients at high risk for cardiovascular morbidity and mortality highlights the need for improved pre-transplant risk stratification. Beyond conventional cardiovascular (CV) risk factors, KT recipients face unique risks stemming from end-stage kidney disease (ESKD) and the effects of immunosuppressive therapy. Existing methods of risk assessment, including standard scoring systems and imaging techniques, have demonstrated suboptimal predictive value for predicting CV events in clinical studies [5, 6]. This shortfall in accurate risk stratification contributes to less effective management and may result in avoidable complications.

The left atrium (LA) plays a central role in cardiac hemodynamics and serves as a sensitive marker of left ventricular (LV) compliance and diastolic performance. While LA volume tends to increase with age, LA emptying fraction remains relatively stable. The LA undergoes both structural and functional remodeling in response to elevated filling pressures, infiltrative diseases, myocardial damage, and arrhythmogenic stimuli [7]. LA function in sinus rhythm can be separated into three components: (1) reservoir function (collection of pulmonary venous blood during LV systole), (2) conduit function (transit of pulmonary venous blood to LV during early LV diastole), and (3) booster pump/contractile function (supplement LV filling during late LV diastole). LA strain (LAS) is a parameter that quantifies myocardial deformation of the LA, providing insight into atrial function and remodeling across the cardiac cycle. It has been demonstrated that LAS is more sensitive than traditional echocardiographic measures in detecting CV pathology in early chronic kidney disease (CKD) as well as ESKD, predicting both diastolic and systolic dysfunction before the appearance of changes in volumes [8, 9]. LAS could also predict major adverse cardiovascular outcomes (MACE) in this population [9]. This predictive ability has been demonstrated in other CV pathologies as well [10].

Despite promising data in CKD and ESKD patients, there is a paucity of data regarding LAS utility in KT recipients. In this study, we sought to investigate the association of LAS with MACE in KT recipients.

## Methods

This was a single-center retrospective study design. We included adult KT recipients at Saint Louis University Hospital between January 1, 2015 and December 31, 2023, who had a pretransplant transthoracic echocardiogram (TTE) available in the hospital cardiac imaging archives. Those TTEs are obtained during routine workup on all patients being evaluated for KT. Patients were excluded if they were not in sinus rhythm at the time of the TTE or if image quality was insufficient for LAS analysis. Variables with more than 20% missing data were excluded from analysis. Patient charts were reviewed for demographic information, clinical comorbidities, laboratory values and medications at the time of listing for KT, as well as pre-transplant cardiac ischemic evaluation and revascularization data. Variable definitions are listed in Appendix 1. Pre-transplant TTE were obtained from the institutional image archive. Twelve investigators performed strain measurements after receiving standardized training from the principal investigator, who completed an advanced cardiac imaging fellowship, to ensure consistent methodology. Interobserver reliability for LAS measurements was assessed using Intraclass Correlation Coefficient (ICC). Before contributing to the study, each investigator was required to demonstrate adequate agreement with the PI’s strain measurements on a set of pre-selected echocardiograms. Interobserver reliability was defined as achieving a limit of agreement (LOA) with the PI of at least 80% on a predefined sample of 10 patients. LA volumes (LAV) were measured using the biplane area–length method and included maximum, minimum, and pre-atrial-contraction volumes calculated using the following formula: (0.848 × area 4-chamber × area 2-chamber)/([length 2-chamber + length 4-chamber]/2). All measured LAV were indexed to body surface area. Using the measured LAV at different points of cardiac cycle, LA function was calculated as follows:

- LA ejection fraction: (LAVmax − LAVmin)/LAVmax.
- Expansion index: (LAVmax − LAVmin)/LAVmin.

While performing measurements, the investigators were blinded to the history of the study subjects and their outcomes. LAS was analyzed using vendor-independent speckle-tracking software (TomTec Imaging Systems GmbH, Unterschleißheim, Germany). LAS was measured from apical four- and two-chamber views. Endocardial borders were automatically traced and manually adjusted as needed to ensure optimal tracking. LAS components were all calculated by averaging the 2 and 4 chamber views.

The primary outcome for the study was the occurrence of MACE, defined as a non-fatal myocardial infarction, unstable angina hospitalization, heart failure hospitalization, fatal arrhythmia, ischemic stroke, or cardiovascular death. Charts were reviewed for outcomes up to March, 2025. Events were identified in the hospital medical records and adjudicated by senior internal medicine residents. In addition to the definitions of each outcome in the data dictionary (appendix 1) created before data collection, specific attention was directed to heart failure hospitalizations to make sure the primary diagnosis was heart failure; evidenced by the workup and the fact that the main management was directed towards heart failure i.e. decongestion, heart failure GDMT, rather than any alternative diagnosis e.g. COPD exacerbation or pneumonia. Patients were divided into two groups according to whether they did or did not have a MACE event (MACE versus NoMACE).

### Statistical analysis

Continuous variables are presented as mean ± SD if normally distributed and as median with interquartile range if not normally distributed. Normality of data was assessed using the Shapiro–Wilk test. Categorical variables are presented as frequencies and percentages. Differences between group mean were evaluated using t tests for continuous variables and χ2 or fisher’s exact test for categorical variables, as appropriate. Kaplan–Meier survival analysis was used to compare MACE-free survival between normal and abnormal LAS, with differences evaluated using the log-rank test. Normal cutoff values used for LASr, LASct, LAScd were 39, 17, and 22, respectively [11]. To evaluate the time-dependent association between LA parameters and the study outcome, Cox Proportional Hazards regression analysis was performed, adjusting for age, and confounding variables identified from univariate logistic regression. To avoid collinearity, LA variables were entered into the model one at a time. All statistical analyses, including multivariable modeling and time-to-event analyses, were performed using Statistical Analysis Software (SAS Institute, Cary, North Carolina, USA).

## Results

Of 518 KT recipients, 377 patients had acceptable quality TTE. Echocardiograms were considered “acceptable” only if the patient was in sinus rhythm during the TTE and if the apical views had adequate image quality for LA wall tracking. Over a mean follow-up duration of 5.3 ± 2.3 years from KT, 82 patients (22%) experienced a MACE event. Baseline demographics, clinical characteristics, laboratory values and medications of the entire study population and both groups at the time of KT are listed in Table 1.

**Table 1:** Patient characteristics

Variable	Total (*n* = 377)	No MACE (*n* = 295)	MACE (*n* = 82)	*P* value
Male, n (%)	214 (56.8%)	167 (56.6%)	47 (57.3%)	0.9
Age, mean ± SD (years)	53.7 ± 13.1	52.9 ± 13.4	56.6 ± 11.4	**0.02**
Race (%)				
White	172 (45.6%)	143 (48.5%)	29 (35.4%)	**0.03**
Black	182 (48.3%)	130 (44.1%)	52 (63.4%)	**0.001**
Other	23 (6.1%)	22 (7.5%)	1 (1.2%)	**0.03**
BMI (kg/m^2^), mean ± SD	29.2 ± 5.7	29.1 ± 5.8	29.6 ± 5.0	0.47
Current or former Smoker (%)	133 (35.3%)	103 (34.9%)	30 (36.6%)	0.77
Hypertension (%)	337 (90.1%)	263 (89.8%)	74 (91.4%)	0.67
Obstructive sleep apnea (%)	110 (29.4%)	83 (28.4%)	27 (32.9%)	0.42
Diabetes mellitus (%)	148 (39.3%)	108 (36.6%)	40 (48.8%)	**0.04**
Coronary artery disease (%)	112 (30.2%)	73 (25.1%)	39 (48.8%)	**< 0.001**
Cerebrovascular accident (%)	13 (3.4%)	8 (2.7%)	5 (6.1%)	0.16
Hemodialysis (%)	226 (65.5%)	165 (61.8%)	61 (78.2%)	**0.007**
Peritoneal dialysis (%)	111 (29.4%)	94 (23.8%)	17 (20.7%)	**0.05**
Dialysis duration (months), mean ± SD	45.6 ± 34.5	41.7 ± 31.0	57.6 ± 41.7	**0.001**
Ischemic evaluation/management (%)				
Nuclear stress test	121 (32.1%)	91 (30.8%)	30 (36.6%)	0.32
Stress echocardiography	254 (68.5%)	203 (70.0%)	51 (63.0%)	0.22
Invasive coronary angiography	14 (3.7%)	12 (4.1%)	2 (2.5%)	0.51
Percutaneous coronary intervention	47 (12.6%)	32 (10.9%)	15 (18.8%)	**0.05**
Laboratory values				
Hemoglobin, mg/dL	10.6 ± 1.7	10.6 ± 1.6	10.5 ± 2.0	0.89
Glycosylated hemoglobin, %	5.6 ± 1.0	5.5 ± 1.0	5.9 ± 1.2	**0.01**
Cardiac medications (%)				
ACE inhibitor or ARB	179 (47.5%)	149 (50.5%)	30 (36.6%)	**0.02**
Anticoagulant	34 (6.6%)	27 (6.5%)	7 (6.9%)	0.74
Antiplatelet	27 (7.2%)	18 (6.1%)	9 (11.0%)	0.13
Aspirin	173 (46.6%)	125 (43.3%)	48 (58.5%)	**0.01**
Beta-blocker	247 (65.5%)	194 (65.8%)	53 (64.6%)	0.84
Diuretic	111 (29.4%)	94 (31.9%)	17 (20.7%)	**0.05**
Statin	268 (71.1%)	202 (68.5%)	66 (80.5%)	**0.03**

Abbreviations: *BMI* Body mass index, *LA* Left atrial, *LAV* Left atrial volume, *LASr* Left atrial strain reservoir, *LASct* Left atrial strain contractile, *LAScd* Left atrial strain conduit.

Statistically significant *P* values are in bold

The mean age was 53.7 ± 13.1 years; with 56.7% male. Patients who experienced MACE were significantly older (56.0 ± 12.2 vs. 52.2 ± 13.3 years, *p* = 0.009) and more likely to be Black (59.8 vs. 44.5%, *p* = 0.005). Patients who developed MACE also had higher rates of diabetes mellitus (46.1 vs. 34.1%, *p* = 0.02), coronary artery disease (48.5% vs. 25.1%, *p* < 0.0001), and prior cerebrovascular incidents (7.8 vs. 2.9%, *p* = 0.038), before KT. They also had longer dialysis duration (56.7 ± 40.7 vs. 44.2 ± 35.5 months, *p* = 0.009), HbA1c (5.9 ± 1.2 vs. 5.5 ± 0.9, *p* = 0.005), and troponin levels (1.5 ± 7.3 vs. 0.1 ± 0.3, *p* = 0.045).

The mean time between pretransplant TTE and KT was 5.2 ± 2.9 months. Table 2 lists the echocardiographic features of patients including LAV and strain measurements. All LAS components were significantly lower in the MACE group. The ICC for strain measurements was 0.96 (95% CI 0.90–0.99). Aortic valve regurgitation severity also differed significantly between groups, with higher rates of mild regurgitation and lower rate of non/trace regurgitation in patients with MACE. LVEF, however, was not significantly different between the two groups.

**Table 2:** Echocardiographic findings

Variable	Total (*n* = 377)	No MACE (*n* = 295)	MACE (*n* = 82)		*P* value
Left ventricular ejection fraction	62.7 ± 8.1	63.0 ± 8.0	61.5 ± 8.7	0.14	
Indexed LVED volume, mL/m^2^	64.5 ± 25.6	64.3 ± 25.5	65.9 ± 27.0	0.75	
Indexed LVES volume, mL/m^2^	23.4 ± 11.9	23.3 ± 11.8	23.9 ± 12.5	0.79	
Left ventricular mass, gm	129.5 ± 81.1	129.3 ± 84.5	130.1 ± 65.7	0.94	
Indexed LV mass (gm/m^2^)	65.3 ± 46.5	65.7 ± 49.7	64.0 ± 30.9	0.79	
Mitral valve regurgitation					
None or trace (%)	237 (65.5%)	191 (64.7%)	46 (56.1%)	0.12	
Mild (%)	110 (30.4%)	82 (27.8%)	28 (34.1%)	0.26	
Moderate (%)	15 (4.1%)	10 (3.4%)	5 (6.1%)	0.33	
Aortic valve regurgitation					
None or trace (%)	305 (84.7%)	246 (83.4%)	59 (72.0%)	**0.01**	
Mild (%)	51 (14.2%)	34 (11.5%)	17 (20.7%)	**0.02**	
Moderate (%)	4 (1.1%)	2 (0.7%)	2 (2.4%)	0.2	
Aortic valve stenosis					
None or trace (%)	340 (96.0%)	266 (90.2%)	74 (90.2%)	0.74	
Mild (%)	11 (3.1%)	9 (3.1%)	2 (2.4%)	1	
Moderate (%)	3 (0.8%)	3 (1.0%)	0 (0.0%)	1	
Maximal LAV, mL	287.8 ± 1628	258.0 ± 1537	395.3 ± 1929	0.52	
Indexed maximal LAV, mL/m^2^	150.4 ± 847.2	129.2 ± 755	225.6 ± 1117	0.39	
Minimal LAV, mL	110.3 ± 673.3	90.3 ± 587	176.8 ± 903	0.34	
Indexed minimal LAV, mL/m^2^	58.0 ± 350.8	44.5 ± 273	102.1 ± 530	0.23	
LA emptying fraction, %	60.5 ± 53.2	59.6 ± 59.9	63.8 ± 14.2	0.57	
LA expansion index	243.9 ± 274.8	241.6 ± 262	252.1 ± 315	0.78	
LA strain (%)					
LASr	30.3 ± 11.1	31.3 ± 11.1	26.5 ± 10.1	**< 0.001**	
LASct	15.2 ± 7.7	15.6 ± 8.1	13.7 ± 5.8	**0.0498**	
LAScd	16.0 ± 7.6	16.8 ± 7.7	13.2 ± 6.3	**< 0.001**	

*BMI* body mass index, *LA* left atrial, *LAV* left atrial volume, *LASr* left atrial strain reservoir, *LASct* left atrial strain contractile, *LAScd* left atrial strain conduit, *LVED* left ventricular end diastolic, *LVES* left ventricular end systolic

Statistically significant *P* values are in bold

Patient outcomes are listed in table 3, stratified by normal versus abnormal LASr. Patients with abnormal LASr had significantly higher MACE events at one, three and five years after KT.

**Table 3:** Cumulative incidence (%) of major adverse cardiovascular events (MACE) following kidney transplant in normal left atrial reservoir strain (LASr) versus abnormal strain (LASr) groups

	Normal LASr			Abnormal LASr			*P* value
Outcome	1 yr	3 yr	5 yr	1 yr	3 yr	5 yr	
Cardiovascular death	0 (0 -3.8)	1.45 (1.4–1.5)	2.9 (2.8- 3.0)	0.34 (0.34–0.34)	2.0 (1.98–2.1)	3.9 (3.8-4.0)	0.84
Non-fatal myocardial infarction	0 (0 -3.8)	0 (0 -4.4)	4.0 (3.8–4.3)	1.4 (1.38–1.42)	3.5 (3.4–3.6)	5.8 (5.6- 6.0)	0.22
Ischemic stroke	0 (0-3.80)	0 (0-4.40)	0 (0-8.50)	0.69 (0.68–0.70)	1.09 (1.08–1.10)	1.62 (1.59–1.65)	**0.04**
Hemorrhagic stroke	0 (0-3.80)	1.45 (1.41–1.49)	1.45 (1.41–1.49)	0.75 (0.73–0.76)	2.46 (2.39–2.52)	2.46 (2.39–2.52)	0.87
Unstable angina	0 (0-3.80)	0 (0-4.40)	0 (0-8.50)	0 (0–1)	2.37 (2.33–2.41)	3.31 (3.24–3.38)	**0.05**
Need for revascularization	0 (0-3.8)	0 (0-4.4)	0 (0-8.5)	0.7 (0.69–0.71)	2.23 (2.19–2.27)	3.7 (3.6–3.8)	0.06
Heart failure hospitalization	0 (0-3.80)	1.47 (1.43–1.51)	6.4 (5.9–6.8)	3.8 (3.7–3.9)	8.3 (8.0-8.5)	10.4 (10.0-10.8)	0.10
Major arrhythmias	0 (0-3.90)	0 (0-4.50)	2.5 (2.4–2.6)	0.73 (0.72–0.74)	1.13 (1.12–1.14)	1.13 (1.12–1.14)	0.88
Total	0 (0-3.80)	4.4 (4.2–4.6)	14.5 (13.3–15.8)	6.2 (6.1–6.4)	16.5 (15.9–17.2)	24.0 (22.8–25.1)	**0.01**

Statistically significant *P* values are in bold

**Fig. 1:**
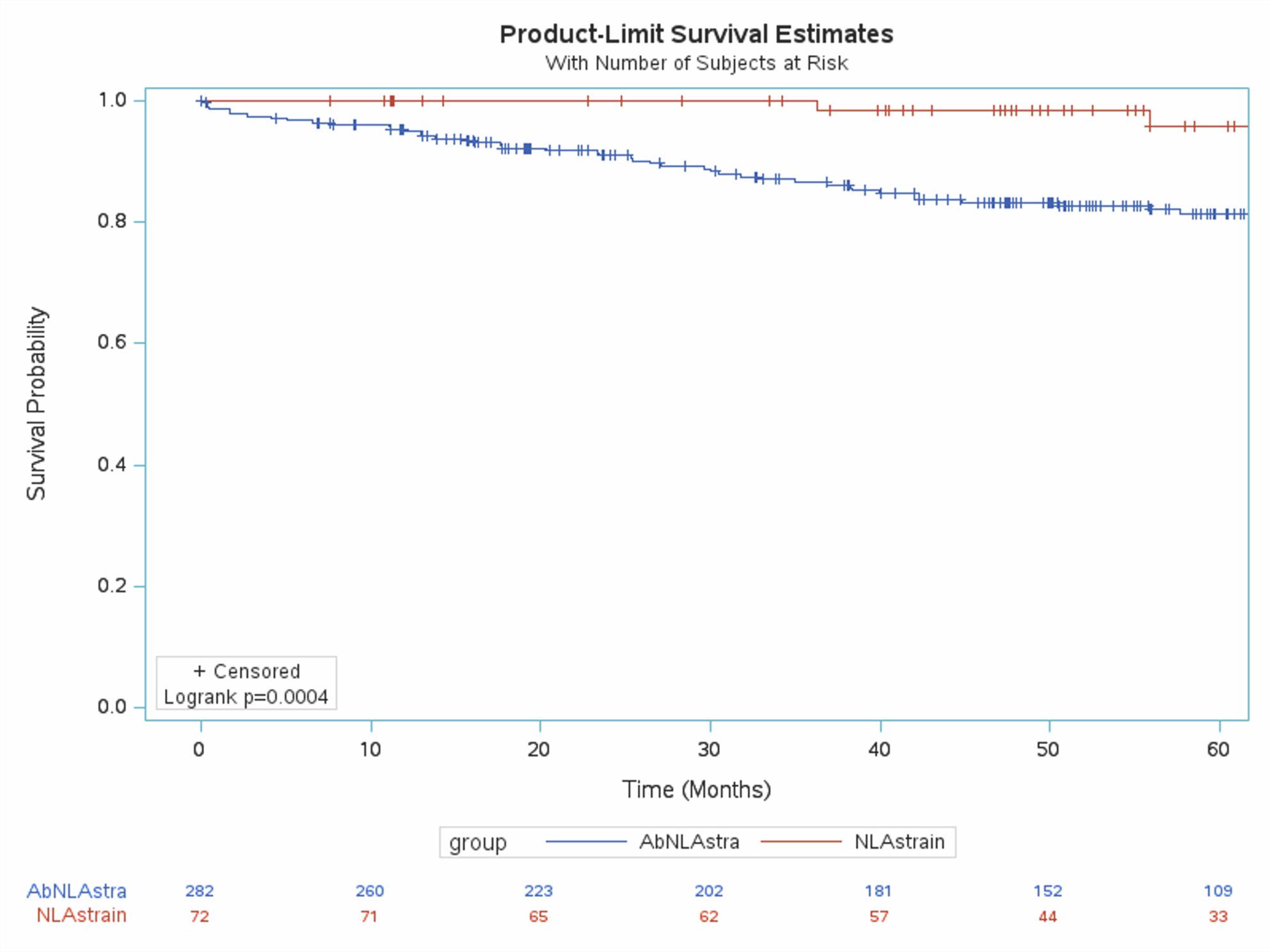
Kaplan-Meir curves in patients with normal vs abnormal left atrial reservoir strain (Normal ≥ 39)

**Fig. 2:**
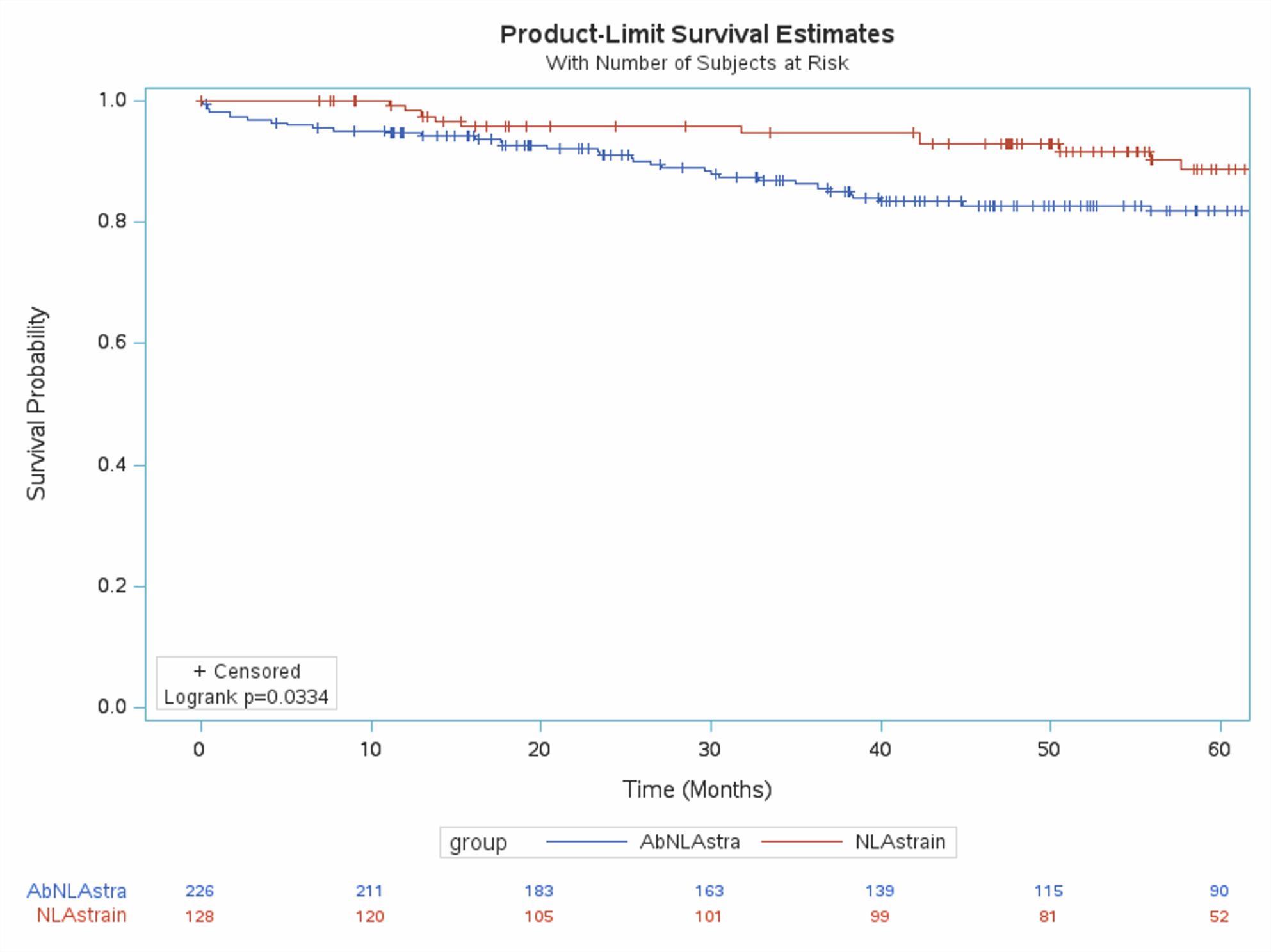
Kaplan-Meier curves in patients with normal vs abnormal left atrial conduit strain (Normal ≥ 22)

**Fig. 3:**
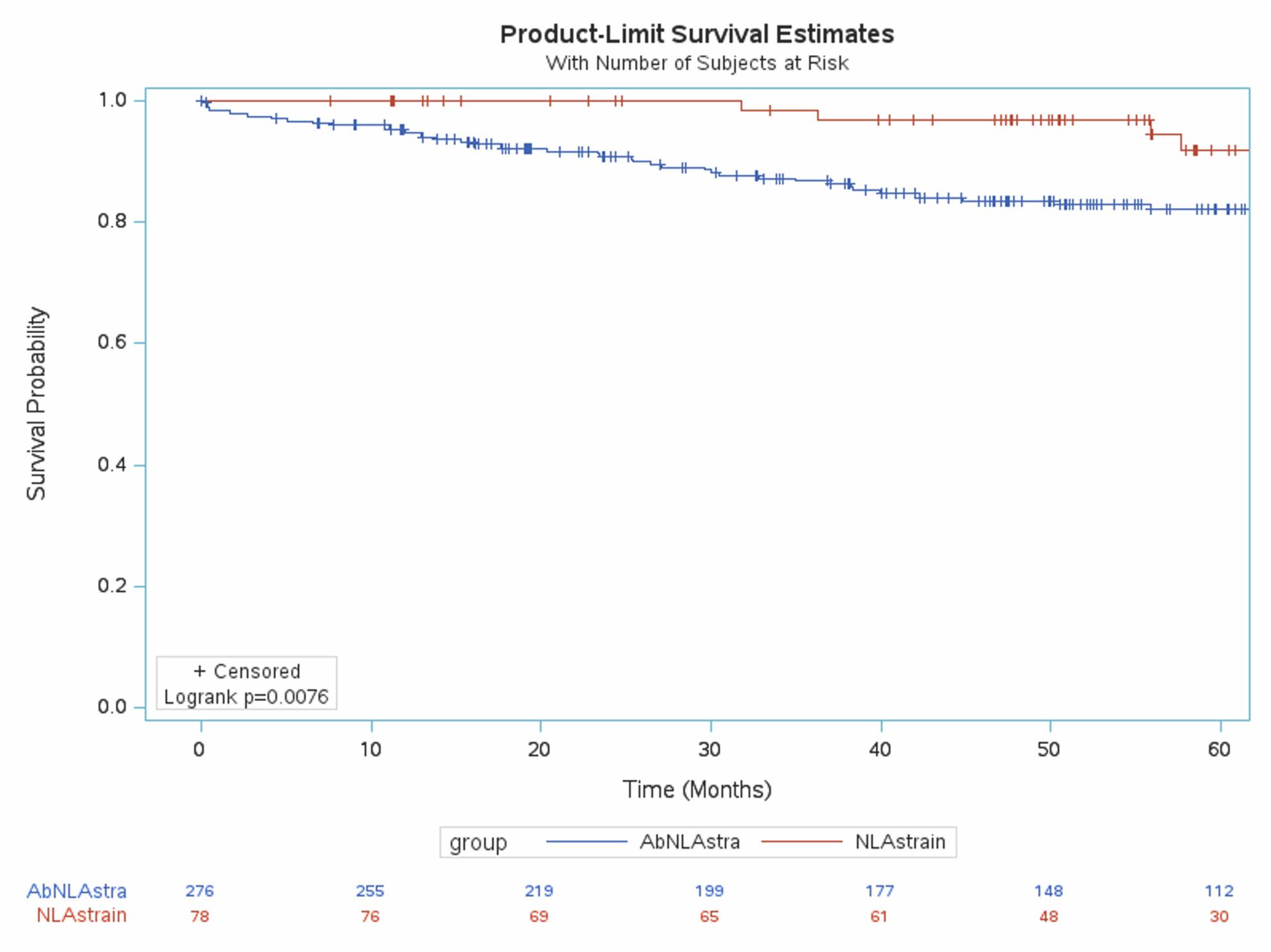
Kaplan-Meier curves in patients with normal vs abnormal left atrial contractile strain (Normal ≥ 17)

Kaplan-Meier curves (Figures 1, 2, 3) showed significantly reduced MACE-free survival in patients with abnormalities in all three LAS components. Confounding variables were identified using univariate regression. Results of univariate regression are provided in Appendix 2. The Cox proportional hazards model was adjusted for statistically significant variables from univariate regression in addition to gender and left ventricular ejection fraction, due to their clinical importance. 

In the Cox model (Table 4), lower LAScd (HR 0.94, 95% CI 0.89–0.98, *p* = 0.003), and LASr (HR 0.97, 95% CI 0.94–0.995, *p* = 0.02) were independently associated with MACE while LASct was not. Other LA function parameters, entered into the model one at a time, were not significantly associated with MACE. The proportional hazards assumption was tested and not violated.

**Table 4:** Cox proportional hazard regression for major adverse cardiovascular outcomes in kidney transplant patients

Variable	Adjusted Hazards Ratio	Confidence Interval	*P* Value
Age	1.00	0.98–1.03	0.69
Gender	1.34	0.76–2.38	0.3
Left ventricular ejection fraction	0.99	0.95–1.02	0.64
Diabetes Mellitus	1.54	0.85–2.79	0.14
Major arrhythmias	1.13	0.38–3.3	0.82
Coronary artery disease	2.31	1.3–4.1	**0.004**
Indexed maximum LAV	1.01	0.99–1.03	0.19
Indexed minimum LAV	1.02	0.98–1.07	0.24
LA emptying fraction	0.97	0.95-1.00	0.06
LA expansion index	1	0.99-1.00	0.75
LASr	0.96	0.94–0.99	**0.01**
LASct	0.96	0.91-1	0.08
LAScd	0.93	0.89–0.97	**0.002**

Adjusted for age, gender, diabetes mellitus, coronary artery disease, arrhythmia and ejection fraction

*LA* Left atrial, *LAV* Left atrial volume, *LASr* Left atrial strain reservoir, *LASct* Left atrial strain contractile, *LAScd* Left atrial strain conduit

Statistically significant *P* values are in bold

## Discussion

In this single-center retrospective study of KTRs, we found that pre-transplant LAS, particularly LASr, and LAScd were independently associated with MACE following KT.

LAS is a reproducible, easy to measure, noninvasive marker of atrial function, compliance and left ventricular diastolic function. LAS may offer additional prognostic information, above the conventional echocardiographic metrics like ejection fraction and Doppler markers of diastolic function, by capturing subclinical myocardial dysfunction. Our results align with previous studies in several populations with cardiovascular disease, including heart failure with preserved ejection fraction, atrial fibrillation, and cardiomyopathy, where impaired LAS has been linked to adverse outcomes [12–14]. KTRs are a unique population in whom both traditional cardiovascular risk factors and kidney-specific factors (e.g., volume overload, dialysis vintage, vascular stiffness) contribute to atrial remodeling [15–20]. From a clinical standpoint, LAS assessment could augment pre-transplant cardiac risk stratification by identifying high-risk patients who may benefit from closer monitoring, early intervention, or tailored cardiovascular management. Our study also confirms previously reported clinical risk factors for post-transplant MACE, including older age, diabetes, and coronary artery disease [21, 22]. Interestingly, left ventricular ejection fraction was not significantly associated with MACE in our cohort, this is counterintuitive. This is likely because the majority of subjects in our study had a preserved systolic function. This limited variability likely reduced the discriminatory ability of the left ventricular ejection fraction. Additionally, LVEF is inherently load-dependent and has poor sensitivity for detecting subclinical myocardial dysfunction and diastolic abnormalities. Finally, in the context of ESRD's unique cardiovascular dynamics, the reversibility of uremic cardiomyopathy post-transplant, might also explain why the ejection fraction was not significantly associated with the long-term outcomes in this specific population. Several limitations of our study should be noted. This was a retrospective single-center study, which may limit generalizability. While our strain analysis was standardized, some inter-institutional variability would likely exist as LAS is fairly operator- and software-dependent. Although our inter-reader reliability was very good, the use of a relatively large number of readers can introduce some subtle variability in measurements. Ascertainment of outcomes relied on chart review, raising the possibility that MACE events were undercounted if patients were hospitalized at outside institutions not captured in our medical record. We did not include data on mitral stenosis severity, which, if present, could have affected LAS values. Finally, despite adjustment for other known confounders, residual confounding from unmeasured variables cannot be excluded.

In conclusion, in this retrospective study, LA strain was independently associated with adverse cardiovascular outcomes in KTRs. Further studies are warranted for external validation of our study findings.

## Supplementary Information

Supplementary Material 1.

Supplementary Material 2.

Supplementary Material 3.

## Data Availability

The datasets used and/or analysed during the current study are available from the corresponding author on reasonable request.

## Abbreviations

CKD: Chronic kidney disease
CV: Cardiovascular
CVD: Cardiovascular disease
ESRD: End-stage renal disease
HF: Heart failure
KT: Kidney transplant
LA: Left atrial
LAScd: Left atrial conduit strain
LASct: Left atrial strain contractile
LASr: Left atrial reservoir strain
LAV: Left atrial volume
LOA: Limit of agreement
LV: Left ventricular
MACE: Major adverse cardiovascular events
TTE: Transthoracic echocardiogram
