# Supplementary material for: Association of left atrial volume and function parameters with cardiovascular outcomes following kidney transplantation
Source: Cardiovasc Ultrasound. 2026 Mar 2;24:7. doi: 10.1186/s12947-026-00369-3 (PMC12951988; doi:10.1186/s12947-026-00369-3)
Supplement: Supplementary file 2 — Supplementary Material 2. [file 12947_2026_369_MOESM2_ESM.docx]

**Appendix 2**

Table: Univariate logistic regression of the association of left atrial function and strain variables with major adverse cardiovascular outcomes in kidney transplant recipients

| **Variable** | **Hazards Ratio** | **95% CI** | **P value** |
| --- | --- | --- | --- |
| **Age(years)** | 1.02 | 1 - 1.04 | **0.01** |
| **Male** | 0.97 | 0.59 - 1.59 | 0.9 |
| **Race** |  |  | 0.32 |
| **Smoking** | 1.07 | 0.64-1.79 | 0.78 |
| **Diabetes mellitus** | 1.65 | 1.01 - 2.71 | **0.04** |
| Major **arrhythmia** | 2.87 | 0.96 - 8.55 | 0.057 |
| **Coronary artery disease** | 2.86 | 1.71 - 4.78 | **<0.001** |
| **Prior PCI** | 1.87 | 0.95-3.66 | 0.06 |
| **Prior CABG** | 0.59 | 0.13-2.73 | 0.5 |
| **Non-fatal MI** | 1.48 | 0.46-4.73 | 0.5 |
| **Hypertension** | 1.31 | 0.56-3.05 | 0.52 |
| **Obstructive sleep apnea** | 1.23 | 0.73-2.09 | 0.42 |
| **Ischemic Stroke** | 2.54 | 0.69–9.24 | 0.15 |
| **Hemorrhagic stroke** | 3.71 | 0.51-26.81 | 0.19 |
| **Prior stroke** | 2.36 | 0.75-7.44 | 0.14 |
| **Heart failure** | 2.05 | 0.91-4.61 | 0.08 |
| **Indexed LVEDV (mL/m²)** | 1 | 0.98–1.02 | 0.76 |
| **Indexed LVESV (mL/m²)** | 0.99 | 0.96–1.03 | 0.92 |
| **LA Volume (mL)** | 1 | 0.99-1.01 | 0.21 |
| **AS severity** |  | | 0.96 |
| **AR severity** |  | | **0.05** |
| **MR severity** |  | | 0.43 |
| **LAEF** | 1.00 | 0.99-1.01 | 0.6106 |
| **LAEI** | 1.00 | 0.99-1.00 | 0.79 |
| **LV ejection fraction (%)** | 0.97 | 0.95-1.01 | 0.17 |
| **LA Reservoir Strain** | 0.96 | 0.93- 0.98 | **<0.001** |
| **LA Conduit Strain** | 0.92 | 0.89- 0.96 | **<0.001** |
| **LA Contractile Strain** | 0.95 | 0.91-0.99 | **0.04** |

Abbreviations: LV, left ventricular; GLS, global longitudinal strain; HF, heart failure; LVEDV, left ventricular end-diastolic volume; LVESV, left ventricular end-systolic volume; LV mass, left ventricular mass; LA volume, left atrial volume; AR, aortic regurgitation; MI, myocardial infarction; MR, mitral regurgitation; PCI, percutaneous coronary intervention; CABG, coronary artery bypass grafting.
