# Supplementary figures and images for: Association of left atrial volume and function parameters with cardiovascular outcomes following kidney transplantation
Source: Cardiovasc Ultrasound. 2026 Mar 2;24:7. doi: 10.1186/s12947-026-00369-3 (PMC12951988; doi:10.1186/s12947-026-00369-3)

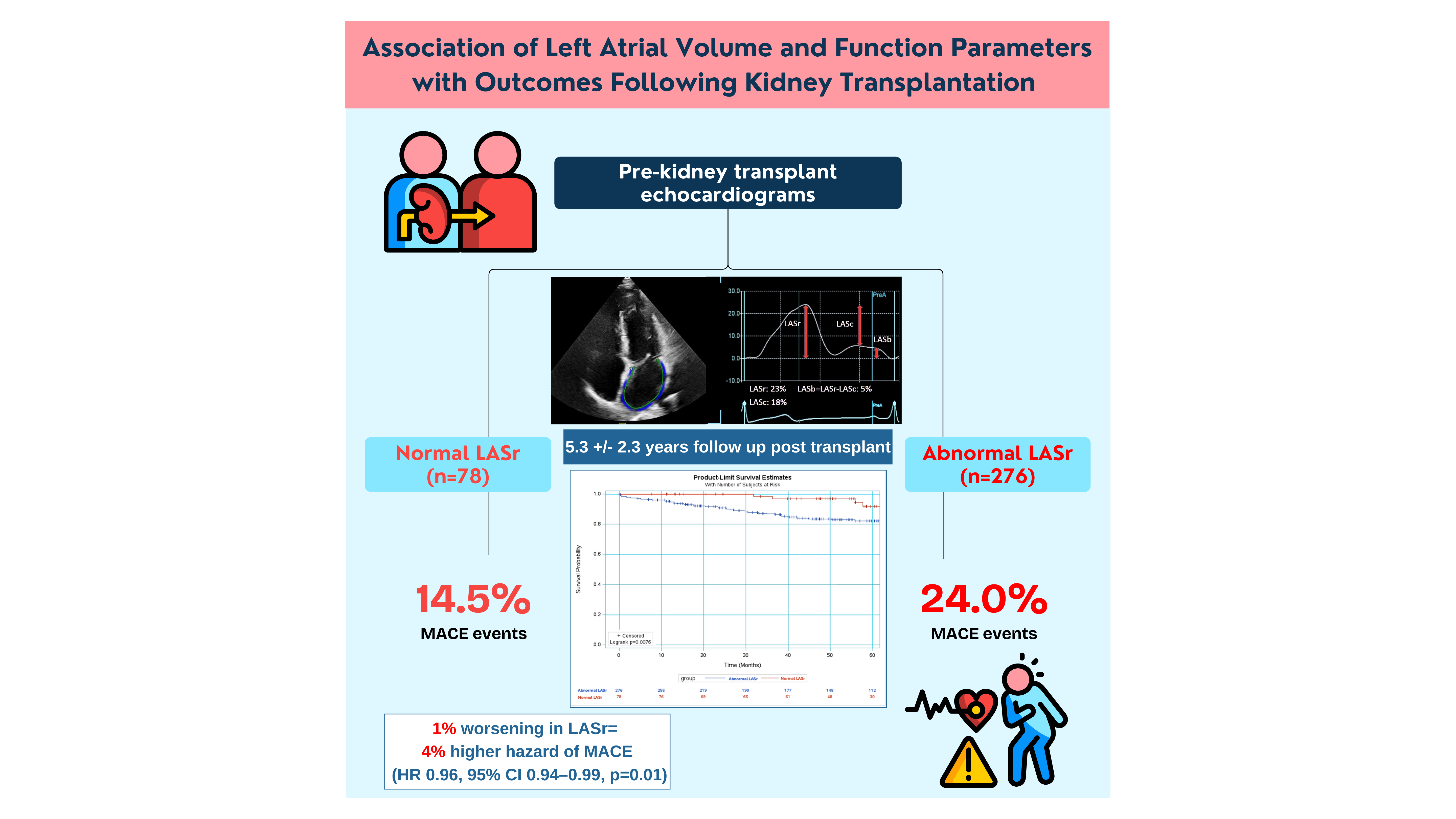

Supplement: Supplementary file 3 — Supplementary Material 3. [file 12947_2026_369_MOESM3_ESM.tiff]
